# Measuring populist discourse using semantic text analysis: a comment

**DOI:** 10.1007/s11135-017-0651-z

**Published:** 2017-11-07

**Authors:** Roel Popping

**Affiliations:** Department of Sociology, University of Groningen, Grote Rozenstraat 31, 9712 TG Groningen, The Netherlands

**Keywords:** Text analysis, Content analysis, Reality claims, Populism

## Abstract

A quantitative index for measuring populist discourse based on the number of times references are made to the own and the other group has been proposed by Aslanidis (Qual Quant, 2017. 10.1007/s11135-017-0517-4). The references to the two groups are found in the actor part of the clauses in texts. In this contribution, it is argued that the clause contains a lot of additional information not on the frequency of occurrence of populist speech, but regarding the how or the why of populist speech. In this text, this is used for a comparison of populist and non-populist statements in speeches by a Prime Minister.

## Introduction

In an interesting article, Aslanidis (2017) proposes to perform research in the field of populism based on text analysis in another way than before. He presents a quantitative index for measuring populist discourse. He discusses the research as was performed in the last years. Main characteristics of populism are the focus on the own group and the other group. Therefore, the occurrence of these two groups in texts is counted. Occurrence of the theme “own group” is counted via the usage of words like ‘we’ and ‘us’ in the text. To indicate the “other group” words in the text like ‘they’ and ‘them’ are counted. The author proposes another method as many occurrences will be detected that should not be counted. Usually a computer analysis is performed based on a dictionary that contains all words or text frames that might occur in a text and that refer to one of the two groups. In this way, all occurrences of the two groups are recorded.

According to the author, investigators should focus on the clauses in texts, especially on the actor part of a clause. This is because actors take action. In case one of the words that might indicate populism is found, a coder has to decide whether this is a case of populist usage or not, and has to code the occurrence as such. Therefore, if for example the word ‘we’ is found in the actor part of the clause, the coder codes it as “own group – populist” (“we have to maintain our own culture”) or as “own group – non-populist” (“we have to pay taxes”). In the same way a word like ‘they’ might be coded as “other group – populist” (“they decide without consulting us”) or as “other group – non-populist” “they are also people”). The other group is also denoted as the anti-elite. The decision populist or not depends on the investigator, who should have formulated a number of rules that allow the coder to distinguish between the two groups. The decision is performed manually, which means by coders who are trained to apply the just mentioned rules. Aslanidis also leaves open how the own group and the anti-elite should be distinguished. This too is up to the investigators in concrete projects. He gives a lot of attention to the distinction between the two groups, in line with that what is usually discussed in research on populism based on text analysis. The frequency of occurrence of the two populist groups is combined into an index for measuring populist discourse. In this way it becomes possible to compare (samples of) texts produced by different groups or to consider developments over time.

The above is welcomed as a first step; the amount of populist speech will not be overestimated anymore. Yet, the author might have gone further. He should not only focus on the amount of populist speech in texts, but also on the how and the why of such speech. The populist speech found in the actor part of the clauses in texts is relevant. Aslanidis starts his plea for considering the clause by a reference to Popping and Roberts (2015). These authors also focus very much on the predicate part of the clause, especially on the verb. Research on populism can also benefit from this information. Hereafter it will be explained how this might be done and what research questions can be answered now. In his text, Aslanidis focuses on populist manifestos. However, more types of text can be used. In the context of research on populism pamphlets and (transcripts) of speeches are important ones. In the example below, data from speeches will be used.

## Elaboration

Although there has been a lively debate and disagreement between scholars on the definition of ‘populism’ (Mudde 2004; Pappas 2016), one of the most general features almost everyone accepts as characteristic to the phenomenon is the permanent referencing to ‘the people’. According to Müller populists do position themselves as the only legitimate representatives of the general will, and that is why they often refer to this will’s very source: the people (Müller 2016). Here populism is considered as a specific type of anti-elite discourse in the name of the people. Aslanidis sees this view both conceptually and methodologically as the most coherent and useful way to understand the phenomenon. In most studies, so far however the focus is on measuring the degree of populism, most of all among political parties (Pauwels 2011; Rooduijn and Pauwels 2011). These investigators only look at the occurrence of populist themes.

Aslanidis claims that investigators should look at information in clauses in sentences in texts. A clause is a sentence or part of a sentence that (explicitly or implicitly) contains an inflected verb, an optional subject and/or object, plus all modifiers related to this verb, subject, and object. The verb actually might consist of two parts: the ‘action’, the activity under consideration, and the ‘position’, the position regarding the activity by the subject. Commonly the subject here is a person, a group of persons or an institution represented by persons. It is not an arbitrary subject, certainly not a ‘thing’. Therefore, researchers often indicate the subject as ‘actor’ or ‘agent’, the initiator of an activity. The ‘object’ is the target of the activity. Positions can only be taken by intentional actors.

In the view of Aslanidis attention is asked for the own group and the anti-elite as specific types of agents. Reference to these types can be in the context of populism or not. So far considering clauses is hardly done in the field of research on populism. Aslanidis refers to Caiania and della Porta (2011) as an exception. Regrettably, he gives no attention to how these investigators use clauses otherwise than that they use a story grammar. He only points to the relevance of the actor, but does not consider the action and the object. Caiania and della Porta actually distinguish three groups. They report that people are ‘betrayed’, ‘neglected’, ‘not respected’, and so on. With regard to politicians, the anti-elite, it is found that these ‘break laws systematically’, ‘tell lies’, ‘latently dissolve democracy’, ‘destroy the country systematically’, and the like. The extreme right people themselves (us, the third group) among others do ‘defend the interests of Germans/Italians’ (note, these are the two groups that are investigated), ‘interpret the will’, ‘fight against the arrogance of power’, ‘criticize’, ‘win the hearts’, ‘will fight for the common good’. These qualifications, which are mostly found in the object part of the clause, are not categorized by the investigators. One might question whether the clause is really used, but anyway the study is a start of making visible what the people and the anti-elites are doing. Most statements that are found contain descriptions of what is going on, sometimes they contain an evaluation.

The three groups above can be the basis for a more detailed coding system. The start for such a system might be found in considering the intended meaning of the clauses in a text in which one of the groups is found. The intended meaning is to describe or judge a process or a state-of-affairs (Roberts 1989). Only by coding this one already gets additional information about each of the groups.

## The reality claim in a clause

As mentioned, Aslanidis starts his plea for considering the clause by a reference to Popping and Roberts (2015). However, he focuses almost exclusively on the actor part of the clause and forgets about the other parts. Hereafter I will do this in line with the authors just mentioned.

According to these authors, clauses might contain reality claims. A reality claim is found in the modal auxiliary verb in a clause, the author of a pamphlet or speech proposes an action by some human actor that has not been performed yet and perhaps will never become more than a proposal or suggestion, but that might shape the audience’s reality. This is also true for populist texts. The semantic grammar used in a reality claim has always two parts at its core, a modal form and its associated rationale. Four types of modal forms are distinguished: *possibility*, for example as expressed in “people can do that,” *impossibility*, as in “people cannot do that,” *inevitability* or *necessity*, as in “people must do that,” and *contingency* or *unnecessity*, as in “people are not compelled do that.” The rationale informs on the reason why. Through modal usage, a text’s source (i.e., its author or speaker) socially constructs what constitutes the possible, the impossible, the inevitable, and the contingent regarding the actor-action-object link. These usages are not intended to convey facts or to describe events; they are used to communicate something about the likelihood of the actor-action-object link. Reality claims are explained in more detail in Roberts et al. (2010), applications are found in among others Popping and Roberts (2015). Developments in text analysis in general are discussed in Popping (2017).

Hereafter a possible start is made with elaborating the use of reality claims in research on populism.

## The rationale in a clause

The rationales provide reasons why something is possible, impossible, and so on. Usually these reasons are framed as *political*, *economic*, *cultural*, *welfare* or *security* related. A motivation for these five rationales is presented in Roberts (2016).

Populists often emphasize nationalism. In this context issues as ‘democratic mandate’ and ‘taking back power’ are examples of political oriented issues; ‘economic forces’ and ‘economic factors’ are examples of economic oriented issues. ‘National freedom fights’ might have to do with political, economic and cultural oriented issues. These are not reasons. Exact reasons should become visible from the context in which the clause containing the saying is used, not from the predicate part in the clause.

Another populist issue today concerns opposition against immigrants, those who are not a member of the own group and seem to be welcomed by the governments, the anti-elite. The reasons for this opposition might be cultural (“…, because they are so different”), welfare (“…, because they take our jobs”), security related (“… because they do badly to our children”). All these arguments might be elaborated into a well-structured classification system that starts from the reasons that were mentioned first. The argument does not have to be correct. It is the view expressed by the speaker.

## Illustrations of reality claim and rationale in a clause

To illustrate reality claims and rationales some sentences are presented taken from speeches by the Hungarian Prime Minister Orbán, who is in office since 2010. Orbán has widely been recognized as a *par excellence* populist (Pappas 2014). Orbán very repeatedly uses populist statements in his speeches. He especially does so when he refers to the Hungarian people as the own group. Sometimes however this group is constituted by the people of countries belonging to the European Union or worldwide. The ‘we’ however might also be reserved for the Hungarian government or the European Union. In some speeches, the European Union is seen as the anti-elite. The reference to people-centrism or anti-elite in the actor part of the clauses in the sentences that are presented should be in the populist sense as suggested by Aslanidis. The rules followed in coding the reality claims and the rationales are found in Popping and Roberts (2009).

Note, these are speeches by the Prime Minister (hereafter PM), sometimes he has an audience where populist remarks can be made easily, for example in a speech to the own people. It can also be that such is not or hardly possible, as in a speech to the PM of another country.

It is absolutely necessary {inevitable} that we {Hungarian people} all take care of everything and everyone that we have been entrusted with: our children, our families, our street, our town, the land that we live in {welfare reason}. (Speech on the celebration of National Day, Budapest, July 1, 2010)

At issue is why the taking care of is inevitable, not what is inevitably to be taken care of. Should care be taken of these things to maintain Hungarians’ quality of life (welfare)? Or to retain things that are essentially Hungarian, like customs and appearance (cultural)? The next sentences in the speech show that the first interpretation is at issue, so the sentence says that there is a welfare reason why something is inevitable for the Hungarian people.

We {Hungarian people} must not {impossible} allow what the people of Hungary have labored so hard to achieve to be taken away again {welfare reason}. We {Hungarian people} must not {impossible} allow the reduction in public utility prices, the tax benefit for families with children, the increase in wages, the family allowance extra scheme, the support provided to mothers and the security of old age to be taken away {welfare reason}. (State of the Nation Address, February 16, 2014)

The rationale for the first sentence is suggested in the next one: Permission to take away achievements is impossible, because these achievements ensure citizens’ welfare. The rational for the second sentence must explain why permission to reduce welfare benefits is impossible. Later in the text, it is explained that in the past few years the Hungarians have been fighting to achieve this and that that was right, a welfare reason.

We {Hungarian people} cannot {impossible} afford to allow Brussels to place itself above the law. We {Hungarian people} cannot {impossible} afford to allow the consequences of madcap policies to be expanded into those countries, which have complied with every treaty and every law – as we have done. We {Hungarian people} cannot {impossible} afford to allow them to force us or anyone else to import the bitter fruits of their misguided policies. We do not want to – and we shall not – import crime, terrorism, homophobia and anti-Semitism to Hungary. {security reason [for all three claims of impossibility]}. (State of the Nation Address, February 28, 2016)

There is a political predicate/action here, but each time the modal clause’s rational is security. The rationale is: we “do not want to import crime, terrorism, homophobia and anti-Semitism to Hungary.”

If we want to stop this mass migration, we {Hungarian people} must {inevitability} first of all curb Brussels {security reason}. The main danger to Europe’s future does not come from those who want to come here, but from Brussels’ fanatics of internationalism. We {Hungarian people} cannot {impossible} allow Brussels to place itself above the law {security reason}. We {Hungarian people} shall not allow {impossible} it to force upon us the bitter fruit of its cosmopolitan immigration policy {security reason}. We shall not import to Hungary crime, terrorism, homophobia and synagogue-burning anti-Semitism. There shall be no urban districts beyond the reach of the law, there shall be no mass disorder or immigrant riots here and there shall be no gangs hunting down our women and daughters. We {Hungarian people} shall not allow {impossible} others to tell us whom we can {no reality claim} let into our home and country, whom we will live alongside, and whom we will share our country with. We know how these things go. First we allow them to tell us whom we must {no reality claim} take in, then they force us to serve foreigners in our country {political reason}. (Speech on 15 March, March 15, 2016)

The first sentence is read as ‘we must curb Brussels because we want to stop migration’. From later context, stopping mass migration means stopping crime, terrorism, etc. So the coding would be “Hungarian people must do something [namely, curb Brussels] for security reasons.” ‘Forcing’ is political. Yet it is the ‘bitter fruit’ of the forcing that hints at the ‘security’ rationale for Hungarians’ making this political-forcing impossible. The previous two sentences provide rationales for sentences 1, 3, and 4. This ‘not allow’ should be coded as ‘impossible’. Subject is ‘Hungarian people’. Rationale is ‘if we allow Brussels to tell us whom we take in, then Brussels will force us to serve foreigners’, which seems ‘political’. The first ‘can’ is not a reality claim because the modal clause is used as an adjective-phrase, modifying ‘whom’. The last ‘must’ is no reality claim because the modal clause is used as an adjective-phrase, modifying ‘whom’.

## Hypotheses

We can look at the number of times each combination of reality claim and rationale is found. This is in line with Popping and Roberts (2015). More interesting, and in line with Aslanidis, is knowing the ratio between the occurrence of such a combination in case the context indicates populist speech is going on versus the context where this is not the case.

The first hypothesis is that in case of a populist context the rationale most of the time is on culture, welfare and security, while in the other situation it is politics and economics. Second, when the populist context applies most of all claims are of the type impossible or inevitable, in the other situation most of all the claim possible is expected. In the third hypothesis combination of rationale and reality claim are considered. In the populist statements, we find more combinations of culture and possibility or impossibility than in non-populist statements. This also holds for welfare in combination with inevitability and security combined with impossibility. In the non-populist statements the combination of economics and inevitable is found more than in populist statements. The combinations in which politics is involved appear as much in populist statements as in non-populist statements.

The motivation is found in that welfare and security are closer to the people than politics and economics, and therefore are more important for populists. In part, this is not in line with the position taken by populist parties. These parties accuse mainstream parties of cutting and slashing welfare at the expense of the ‘native’ population and to the benefit of the ‘undeserving’ immigrant (Schumacher and Van Kersbergen 2016). However, here sayings by the PM are used, a PM who refers directly to the needs for the Hungarian people.

Populists can better explain why thing have to remain as they are than that they can ask for changes. Therefore the idea of changes are impossible has a lot of sympathy in the group.

## Results

A first analysis of the last three paragraphs, that do not only contain words of thanks, gratitude or wishing success, of the 228 speeches (containing 333 reality claims) by the PM from the start in 2010 until end 2016 show that the reality claims he uses most are inevitability and impossibility. The speeches are found on English version of the government’s website. The website also contains press releases, press conferences and interviews. These however are not used here. Sentences in passive voice are rewritten in active voice.

Aslanidis has not clearly presented rules for deciding on whether or not a populist view applies. He instructed his raters “to read their assigned material at least twice to acquaint themselves with the context. In this first, holistic stage, they were instructed to consider the main subjects addressed by the manifesto and locate emergent collective identities, adversarial or otherwise” (Aslanidis 2017: 16). In line with this finding is the opinion expressed by Hawkins (2009: 1048) that “the label of populist is often applied without any systematic empirical justification.” The words ‘we’ and ‘they’ as actor are used in the speeches. Sometimes however names are used, most of all ‘European Union’. In the present study, where necessary based on context information, it is first decided which of the two groups is considered. Next, it is to be decided whether the statement containing the group reference is populist or not. This also is distinct from the context. In case the homogeneous own group is referred to (‘we Hungarians’, ‘we Europeans’), and it is not an official body like the government, and the statement is not the communication of a fact, this is coded as populist. All neutral expressions or situations in which there is doubt are considered as non-populist. Populist usage is also found in case a homogeneous ‘they’ group that is actor in the clause does bad to the own group. In order to see whether the populist speech is coded in a consistent way the coding is performed a second time by another independent rater. The index Scott’s π (Scott 1955) is used to compute intercoder agreement. In order to have reliable results, indicating that it does not matter which rater performed the assignments the outcome 0.80 is used as lower bound. This value is generally used in case a common coding task is performed. For ‘we’ in a populist sense or not the outcome π = 0.85 (N = 320) is found, and for ‘they’ in the two senses π = 0.83 (N = 13). These outcomes indicate that the coding of populist or not is performed in a reliable way. The total dataset contains 2743 expressions of ‘we’ and 271 expressions of ‘they’. Only clauses containing a reality claim have been coded with regard to use in a populist context or not.

The data are now coded using the following template: According to the PM for {political, economic, welfare, cultural, security} reasons it is {possible, impossible, inevitable, contingent} that a certain someone does as certain something.

Table 1 shows the frequency of occurrence of all combinations of reality claim and rationale in the two types of speech. Note that a number of combinations did not occur at al. In order to answer to the hypotheses odds ratios (Rudas 1998) are used. The outcome 1 for a specific combination indicates that the chance on the occurrence of this combination is the same in the populist statements as in the non-populist statements. An outcome > 1 informs that the chance on the use of the combination is highest in populist statements and the outcome < 1 denotes the reverse. The first order odds ratios for the rationale culture, welfare and security are resp. 3.64, 0.80 and 2.52. For politics and economics, the ratios are 0.41 and 0.05. Except for the rationale welfare this is in line with the first hypothesis. With respect to the reality claims, the ratios for possibility, impossibility and inevitability are resp. 0.81, 2.13 and 0.54. The second hypothesis did not mention inevitability explicitly, this implies it is suggested that the ratio should be around 1. The ratio found is far less. The reality claim contingent is not considered as it only was found once.

**Table 1:** Reality claim by rationale by populist speak

Populist speak	Reality claim	Rationale					
		Politics	Economics	Welfare	Culture	Security	Total
Yes	Possible	1	0	1	2	0	4
	Impossible	4	0	5	13	13	35
	Inevitable	13	1	24	25	15	78
	Contingent	0	0	0	0	0	0
	Total	18	1	30	40	28	117
No	Possible	3	0	4	1	1	9
	Impossible	13	4	11	5	3	36
	Inevitable	50	30	50	20	20	170
	Contingent	0	0	0	1	0	1
	Total	66	34	65	27	24	216

Next it should be considered whether there are combinations of rationale and reality claim which are used more often by the PM in populist statements or just not. Table 2 contains the second order odds ratios. The chance on running into the combinations culture and possible and welfare and inevitable are much higher in populist statements than in non-populist statements. The chance on inevitable and economics is highest in non-populist speech. The chance on possible and politics is about equal in both types of statements. All findings are in line with the assertion in hypothesis 3.

**Table 2:** Second order odds ratios

Reality claim	Rationale				
	Politics	Economics	Welfare	Culture	Security
Possible	1.65	***	0.50	2.27	0.00
Impossible	0.46	0.00	0.37	1.04	3.83
Inevitable	1.74	> 10	2.84	1.02	0.34
Contingent	***	***	***	0.00	***

Some ratios are not be computed as dividing by zero is not possible

In the above, only those statements by the PM are considered in which a reality claim is used. Information is used on whether these statements are used in a populist context or not. Now we have fewer statements left than when the method proposed by Aslanidis is followed, but in our statements an actor is suggested who has to take some action. This makes that we have learned more than when only the amount of ‘we’ or ‘they’ references in a populist context are counted.

The findings can be extended depending on the research question one has. Above it is not considered whether certain uses of rationale, reality claim or combination are changing over time. The suggested actor is not investigated. The actor can be someone from the ‘we’ or the ‘they’ group, but it can also be the Hungarian people, the European people, who are usually presented as part of the ‘we’ group, or the European Union, who is sometimes own group, but at other times really as the anti-elite. In many situations, it might also be useful to consider the predicate, especially the object part of the clause. Anyway one gets far more information than in the situation where only (the occurrence of) themes are counted. Therefore the comparison of different groups or over time can include much more than only considering the amount of populist speech. Above only information taken from speeches by one person was used. There are several sources from which the information can be taken however (pamphlets, manifestos, editorials in newspapers or reports in radio or TV broadcastings) and this also allows for more different research questions.

One must be aware however that in the approach presented above many populist sayings are not included, simply because only clauses containing reality claims are used. This implies that the following sentence: “We will achieve a great and glorious victory.” (Speech: We Ask of Four More Years, March 29, 2014) is not included in the data. Depending on the research question, such sayings (description of a judgment) can be important.

## Discussion

Populism is measured in two different ways. Some investigators look at terminology used, others look at the reference to the ‘we’ and the ‘they’ groups. This second position is taken by Aslanidis. He asked attention for the clauses in the sentences in a text, but he only asked attention for the actor part of such a clause. He needs this in order to develop an index for measuring populist discourse. In this text it is emphasized that one should go further and also should consider the predicate part of the clause. Especially reality claims and rationales found in clauses in texts are very constructive as they can be used to draw inferences about how populists believe that topics that are relevant for them should be addressed. This will enrich the research on populism. An example has been presented to show this all.

Pauwels (2011) is an example of a study in which terminology is used. Populism is based on the occurrence of specific words. This leads to different findings. Out of the 333 clauses used in the present study only 44 contain a populist expression based on the dictionary used by Pauwels (2011: 119). In 6 of these clauses an expression was found twice. 20 of these expression had been coded as populist based on the actor’s context.

The type of documents analyzed might already have a great impact on the approach to be followed in coding. Speeches and debates will contain a lot of ‘we’ and ‘they’ references, but will this also be true for manifestos and party-programs? There concrete issues might be more important to be named. Besides the view on populism by the investigator will also play an important role.

Aslanidis has not presented explicit rules for determining whether the speech is populist or not. We used such rules. But there is room for own interpretations by the raters, see the interrater agreement values found. Therefore the rules need a further discussion, but this discussion should be performed by researchers who really study populism.
